# Screening for asymptomatic coronary artery disease in patients with diabetes mellitus: A systematic review and meta-analysis of randomized trials

**DOI:** 10.1186/s12872-016-0256-9

**Published:** 2016-05-10

**Authors:** Christophe Bauters, Gilles Lemesle

**Affiliations:** Centre Hospitalier Régional et Universitaire de Lille, Lille, France; Inserm U1167, Institut Pasteur de Lille, Université Lille Nord de France, Lille, France; Faculté de Médecine de Lille, Lille, France; Hôpital Cardiologique, CHRU de Lille, Boul. Prof. Leclercq, 59037 Lille Cedex, France

**Keywords:** Diabetes mellitus, Coronary artery disease, Screening, Randomized study, Stress test, Coronary angiography, Percutaneous coronary intervention, Coronary artery bypass surgery

## Abstract

**Background:**

Screening diabetic patients for the presence of asymptomatic coronary artery disease (CAD) may potentially impact therapeutic management and outcome. We performed a systematic review and meta-analysis of randomized trials addressing this question.

**Methods:**

We searched the PubMed database for studies reporting a randomized comparison of systematic screening for CAD in diabetic patients versus no systematic screening. The screening protocols were variable with the use of exercise electrocardiogram test, or stress echocardiography, or nuclear test, or coronary computed tomography angiography.

**Results:**

The final analysis included 5 randomized studies and 3,314 patients altogether. The screening strategy had no detectable impact on outcome with odds ratios (OR) [95 % confidence interval (CI)] of 1.00 [0.67–1.50], 0.72 [0.33–1.57], 0.71 [0.40–1.27], and 0.60 [0.23–1.52] for all-cause death, cardiovascular death, non-fatal myocardial infarction, and the composite cardiovascular death or non-fatal myocardial infarction, respectively. Protocol-related coronary procedures were relatively infrequent in screened patients: coronary angiography was performed in 8 % of the cases, percutaneous coronary intervention in 2.5 %, and coronary artery bypass surgery in 1.5 %. There was no evidence for an effect of screening on the use of statins (OR = 1.19 [0.94–1.51]), aspirin (OR = 1.02 [0.83–1.25]), or angiotensin-converting enzyme inhibitors/angiotensin receptor blockers (OR = 0.97 [0.79–1.19]).

**Conclusion:**

The present analysis shows no evidence for a benefit of screening diabetic patients for the presence of asymptomatic CAD. The proportion of patients who undergo myocardial revascularization as a consequence of screening was low.

**Electronic supplementary material:**

The online version of this article (doi:10.1186/s12872-016-0256-9) contains supplementary material, which is available to authorized users.

## Background

Diabetes mellitus (DM) is a major risk factor for the development of coronary artery disease (CAD) [1]. CAD is frequently asymptomatic in diabetic patients and is often diagnosed when an acute myocardial infarction (MI) occurs [2]; these acute events are frequently responsible of irreversible myocardial damage, even when modern therapeutic strategies are used [3]. Moreover, when the diagnosis of CAD is made, the prognosis of diabetic patients is worse than that of non-diabetic patients [4–7]. Since an early detection of the disease may potentially impact therapeutic strategies and prevent cardiac events, there has for long been interest in the screening of diabetic patients for the presence of asymptomatic CAD [8, 9]. In 2009 however, the report of the Detection of Ischemia in Asymptomatic Diabetics (DIAD) study has modified the perception of the benefit of this approach by showing similar rates of cardiac events in the screening and the control groups [10]. Although DIAD was a breakthrough study with rapid incorporation into the guidelines, it was acknowledged that, due to the low rate of events overall, the power of the study to exclude a difference in outcome was limited. Since then, several other studies randomizing screening strategies in diabetic populations have been published, including very recently [11]. In the present paper, our aim was to perform a systematic review and meta-analysis of randomized trials that addressed this question.

## Methods

### Search strategy

The PubMed database was searched for eligible studies with no restriction of time in January 2016 by using the terms « (((diabetes) AND coronary) AND screening) AND “randomized controlled trial” [Publication Type] » and the filters « Humans » and « English ». Two investigators (CB and GL) independently checked retrieved titles and abstracts for eligibility and relevant full texts were systematically retrieved for further detailed assessment. The search procedure was repeated in Science Direct and ISI Web of Knowledge (All databases). Major reviews regarding screening for CAD in diabetic patients were also hand-searched. Cross-references and quoted papers were checked to identify other relevant studies. The retrieved studies were examined to exclude overlapping data. Unpublished data and meeting abstracts were not considered for the present analysis because they could not provide adequately detailed data and their results might not be final.

### Study eligibility

Studies were eligible only if they reported a randomized comparison of systematic screening for CAD in diabetic patients versus no systematic screening. Inclusion criteria were: *(i)* prospective randomized study; *(ii)* comparison of screening for CAD with exercise electrocardiogram test (ETT), or stress echocardiography, or nuclear stress test, or coronary computed tomography angiography (CCTA), versus no screening; *(iii)* in asymptomatic diabetic patients with no evidence of CAD; *(iv)* with a follow-up > 1 year; (*v*) with availability of at least one of the following events at follow-up: all-cause death, cardiovascular death, non-fatal MI, the composite of cardiovascular death or non-fatal MI. Exclusion criteria were: *(i)* cohort studies; *(ii)* studies performed in patients with prior CAD; *(iii)* studies with overlapping data; *(iv)* studies performed in animals; *(v)* studies performed without final report and only abstracts available.

### Data extraction

The following information was extracted from each study: study name and first author; number of patients; monocentric or multicentric study; geographic area; period of inclusion; duration of follow-up; inclusion criteria; exclusion criteria; screening protocol; treatment plan if screening test was abnormal; baseline characteristics of the patients for the different studies (age, body-mass index, gender, smoking, systolic and diastolic blood pressure, low-density lipoprotein cholesterol, high-density lipoprotein cholesterol, triglycerides, type of DM, duration of DM, insulin treatment, levels of glycated hemoglobin, retinopathy, peripheral vascular disease, statin use at baseline, aspirin use at baseline, angiotensin-converting enzyme inhibitor/angiotensin receptor blocker (ACE/ARB) use at baseline); number of patients with all-cause death, cardiovascular death, non-fatal MI, the composite of cardiovascular death or non-fatal MI, in the screening groups versus the control groups at follow-up; number of patients receiving statins, aspirin, ACE/ARB in the screening groups versus the control groups at follow-up; number of patients with abnormal screening; number of patients with protocol-related coronary procedures (coronary angiography, percutaneous coronary intervention (PCI), coronary artery bypass surgery (CABG)) in the screening group.

### Data synthesis and statistical analysis

Statistical analysis was performed using STATA version 14.0, StataCorps, College Station, Texas. Odds ratios (ORs) and 95 % confidence intervals (CI) of all-cause death, cardiovascular death, non-fatal MI, cardiovascular death or non-fatal MI, statin use, aspirin use, ACE/ARB use were calculated using the *metan* command. Overall estimates of effect were calculated with a random-effects model. Between-study statistical heterogeneity was assessed by using the Cochran Q chi-square and the *I*^2^ test. Small-study effect was assessed visually by examining funnel plots of each trial effect size against the standard error.

## Results

### Search results and study selection

**Fig. 1:**
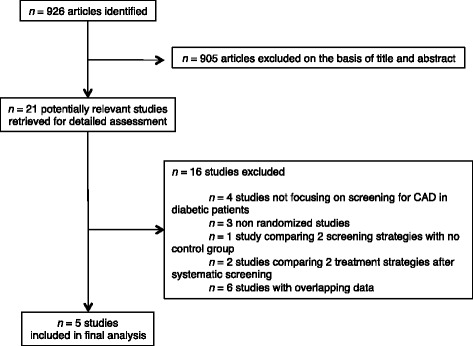
Flow chart of the study selection process

We found 926 citations in PubMed. Among 21 potentially relevant studies for which a detailed assessment of the full-text was performed, 16 were excluded. The reasons for exclusion were: studies not focusing on screening for CAD in diabetic patients (*n* = 4), non randomized studies (*n* = 3), studies comparing two screening strategies with no control group (*n* = 1), studies comparing 2 treatment strategies after systematic screening (*n* = 2), and studies with overlapping data (*n* = 6). We finally included 5 randomized comparisons of systematic screening for CAD in asymptomatic diabetic patients versus no systematic screening: the Faglia et al. study [12], the DIAD study [10], the Do You Need to Assess Myocardial Ischemia in Type-2 diabetes? (DYNAMIT) study [13], the FACTOR-64 study [14], and the Does coronary Atherosclerosis Deserve to be Diagnosed earlY in Diabetic patients? (DADDY-D) study [11]. The search procedure was repeated in ScienceDirect and ISI Web of Knowledge; we found no additional studies that met our inclusion criteria. Further searches using cross-references and quoted papers as well as analyses of review papers on screening for CAD in diabetic patients did not identify other studies. The study selection process is summarized in Fig. 1.

### Studies and patients characteristics

Table 1 shows the characteristics of the five included studies. The number of patients ranged from 141 to 1,123 (total = 3,314). All studies were randomized; recruitment was monocentric in the Faglia et al. and in the DADDY-D studies, and multicentric in DIAD, DYNAMIT, and FACTOR-64. Overall, the inclusion periods varied from 1998 to 2013 and the follow-up time was in the 3 to 5-year range. With the exception of FACTOR-64 in which there was a small proportion (12 %) of patients with type 1 DM, inclusion was limited to patients with type 2 DM. Inclusion criteria are summarized in Table 1. The Faglia et al. and DYNAMIT studies focused on diabetic patients with other risk factors; DADDY-D included diabetic patients with high cardiovascular risk score. The screening protocols were variable with the use of ETT, stress echocardiography, or nuclear stress test to detect myocardial ischemia; in the Faglia at al. study, two tests were used; in FACTOR-64, CCTA was used to directly test for the presence of coronary atherosclerosis rather than assessing ischemia. Treatment plans in case of abnormal screening test were also variable. In Faglia et al., FACTOR-64 and DADDY-D, coronary angiography was recommended, while in DIAD and DYNAMIT, subsequent investigations were left at the decision of the physician or cardiologist. The overall quality assessment of the different studies was good (Additional file 1: Table S1).

**Table 1:** Description of the studies included in the meta-analysis

	Faglia et al. (2005)	DIAD (2009)	DYNAMIT (2011)	FACTOR-64 (2014)	DADDY-D (2015)
Number of patients	141	1123	631	899	520
Design	Randomized	Randomized	Randomized	Randomized	Randomized
	Monocenter	Multicenter	Multicenter	Multicenter	Monocenter
	Italy	USA and Canada	France	USA	Italy
Period of inclusion	July 1998 to July 1999	July 2000 to August 2002	December 2000 to June 2003	July 2007 to May 2013	September 2007 to May 2012
Follow-up	4.3 years	5 years	3.5 years	4 years	3.6 years
Inclusion criteria	- Type 2 DM - 45–75 years - At least 2 of the following: 1. total cholesterol ≥240 mg/dL or HDL-cholesterol ≤35 mg/dL or pharmacological therapy 2. blood pressure >140–90 mmHg or pharmacological therapy 3. active smoking 4. albumin excretion >30 mg/24 h 5. family history of CAD	- Type 2 DM occurring at age 30 years or older with no history of ketoacidosis - 50–75 years	- Type 2 DM - 55–75 years - At least 2 of the following: 1. albumin excretion >30mg/L or >30 mg/24 h 2. treated or untreated hypertension 3. treated or untreated lipid abnormality 4. peripheral arterial disease 5. prior transient ischemic accident 6. smoking 7. family history of CAD	- Type 1 or type 2 DM - Men ≥50 years or women ≥55 years with DM documented for ≥3 years, or men ≥40 years or women ≥45 years with DM documented for ≥5 years - Use of antidiabetic medication for ≥1 year prior to enrollment	- Type 2 DM documented for ≥1 year - 50–70 years - Normal sinus rhythm on ECG - Cardiovascular risk score ≥10 % according to Italian risk chart [11] - Ability to exercise
Exclusion criteria	- Dialysis - Leg amputation - Poor-prognostic disease	- Angina pectoris or chest discomfort - Stress test or coronary angiography within the prior 3 years - History of MI, heart failure, or coronary revascularization - Abnormal results of rest ECG - Any clinical indication for stress testing - Active bronchospasm precluding the use of adenosine - Limited life expectancy due to cancer or end-stage renal or liver disease	- History of MI, CAD, or stroke - Previous positive stress test or myocardial perfusion imaging - Previous negative stress test or myocardial perfusion imaging within the last 3 years	- Any documented atherosclerotic cardiovascular disease - Treatment with an investigational drug within 30 days - Therapy or condition posing a risk for adherence to study requirements - Pregnancy, lactation, or childbearing potential without effective contraception - Limited life expectancy or comorbidity making primary screening and treatment inappropriate	- Prior CAD or heart failure - ETT or other stress testing or coronary angiography performed within 1 year - ECG showing left bundle branch block or ST depression >1 mm or a Q wave - Glomerular filtration rate <40 ml/min/1.73 m^2^ - Contraindication to double antiplatelet agent treatment - Life-threatening condition or major psychiatric disorder or active drug abuse
Screening protocol	ETT and dipyridamole stress echography.	Adenosine Tc-99 m sestamibi myocardial perfusion imaging.	ETT or dipyridamole SPECT in patients unable to perform the exercise test, with a sub-maximal negative exercise test result or with ECG abnormalities impairing the interpretation of the exercise test.	CCTA screening. If the serum creatinine level was 2.0 mg/dL or greater for men or 1.8 mg/dL or greater for women, or if some other contraindication to performing CCTA was present, screening was performed without contrast, and only a CAC score was obtained.	ETT.
Treatment plan if screening test abnormal	All subjects with ≥1 test positive were advised to undergo coronary angiography. All subjects with positive screening had to undergo cardiological consultation and follow-up. All the subjects with negative screening and the subjects in the control arm did not undergo any cardiological workup in the absence of any cardiac symptoms.	None. The results of the screening test were communicated to the participants and their personal physicians.	None. Subsequent investigations (such as coronary angiography) and treatments (such as revascularization procedures) were left at the cardiologist’s decision.	Based on CCTA results, patients with severe stenosis were recommended to undergo coronary angiography; patients with moderate stenosis were recommended to receive stress cardiac imaging. Standard medical management was recommended to patients of the control group and to patients with normal CCTA. Patients with abnormal CCTA or a CAC score >10 were recommended to begin aggressive care to reduce risk factors [14].	Coronary angiography was proposed to all patients with positive ETT.

*CAC* coronary artery calcium, *CAD* coronary artery disease, *CCTA* coronary computed tomography angiography, *DM* diabetes mellitus, *ECG* electrocardiogram, *ETT* exercise electrocardiogram test, *HDL* high-density lipoprotein, *MI* myocardial infarction, *SPECT* single photon emission computed tomography

As shown in Table 2, the mean age varied between 58 and 64 years and the proportion of women varied between 20 % and 48 %. The mean duration of DM ranged from 8 to 13 years and the mean HbA1c from 7.0 to 8.7 %. The distribution of other coronary risk factors among studies is summarized in Table 2 as well as the use of insulin, statins, aspirin and ACE/ARB prior to randomization.

**Table 2:** Description of the baseline characteristics of the patients in the different studies included in the meta-analysis

	Faglia et al. (2005)	DIAD (2009)	DYNAMIT (2011)	FACTOR-64 (2014)	DADDY-D (2015)
Number of patients					
- Screening	71	561	316	452	262
- Control	70	562	315	447	258
Age, y ± SD					
- Screening	58.7 ± 8.3	60.7 ± 6.7	64.1 ± 6.4	61.5 ± 7.9	61.9 ± 4.8
- Control	61.5 ± 8.1	60.8 ± 6.4	63.7 ± 6.4	61.6 ± 8.4	62.0 ± 5.1
BMI, kg/m^2^ ± SD					
- Screening	27.2 ± 5.1	31.1 ± 6.5	30.4 + 4.7	32.9 ± 6.8	29.6 ± 4.9
- Control	28.3 ± 4.6	31.0 + 6.1	30.8 ± 5.3	33.4 ± 7.1	30.6 ± 7.2
Women					
- Screening	39 %	48 %	45 %	48 %	20 %
- Control	47 %	45 %	46 %	47 %	20 %
Smokers^a^					
- Screening	65 %	10 %	17 %	17 %	40 %
- Control	79 %	9 %	15 %	15 %	38 %
Systolic BP, mmHg ± SD					
- Screening	143 ± 19	133 ± 17	–	129 ± 12	140 ± 15
- Control	142 ± 17	132 ± 16	–	131 ± 11	141 ± 15
Diastolic BP, mmHg ± SD					
- Screening	86 ± 11	80 ± 9	–	74 ± 8	82 ± 7
- Control	84 ± 10	79 ± 8	–	74 ± 8	81 ± 7
LDL-C, mg/dL ± SD					
- Screening	–	114 ± 32	–	86 ± 29	125 ± 37
- Control	–	114 ± 33	–	88 ± 33	119 ± 33
HDL-C, mg/dL ± SD					
- Screening	49 ± 15	50 ± 15	–	45 ± 14	42 ± 11
- Control	46 ± 15	49 ± 15	–	45 ± 13	42 ± 12
Triglycerides, mg/dL ± SD or [95 % CI]					
- Screening	154 ± 105	172 ± 118	–	144 [99–201]	163 ± 140
- Control	161 ± 88	168 ± 101	–	132 [92–198]	161 ± 96
Type 2 diabetes					
- Screening	100 %	100 %	100 %	88 %	100 %
- Control	100 %	100 %	100 %	88 %	100 %
Duration of DM, y ± SD					
- Screening	11.6 ± 10.6	8.2 ± 7.1	–	12.3 ± 9.2	9.9 ± 6.7
- Control	11.3 ± 10.3	8.9 ± 6.9	–	13.5 ± 10.7	10.0 ± 6.2
Insulin					
- Screening	11 %	24 %	–	43 %	23 %
- Control	14 %	22 %	–	43 %	21 %
HbA1c, % ± SD					
- Screening	8.6 ± 2.3	7.2 ± 1.6	8.6 ± 2.2	7.4 ± 1.4	7.7 ± 1.4
- Control	8.4 ± 1.9	7.0 ± 1.5	8.7 ± 2.0	7.5 ± 1.4	7.8 ± 3.1
Retinopathy					
- Screening	56 %	14 %	–	–	14 %
- Control	59 %	16 %	–	–	17 %
Peripheral vascular disease					
- Screening	–	9 %	14 %	–	5 %
- Control	–	9 %	14 %	–	7 %
Statins use (baseline)					
- Screening	39 %	37 %	–	77 %	49 %
- Control	30 %	41 %	–	72 %	44 %
Aspirin use (baseline)					
- Screening	13 %	43 %	–	43 %	29 %
- Control	17 %	46 %	–	41 %	26 %
ACE/ARB use (baseline)					
- Screening	14 %	37 %	–	–	62 %
- Control	14 %	41 %	–	–	65 %

*ACE/ARB* angiotensin-converting enzyme inhibitors/angiotensin receptor blockers, *BMI* body-mass index, *BP* blood pressure, *CI* confidence interval, *DM* diabetes mellitus, *HbA1c* glycated hemoglobin, *HDL-C* high-density lipoprotein cholesterol, *LDL-C* low-density lipoprotein cholesterol

^a^data are for smokers in Faglia et al. and DYNAMIT, current smokers in DIAD and DADDY-D, former or current smokers in FACTOR-64

### Effect of screening on clinical events during follow-up

**Fig. 2:**
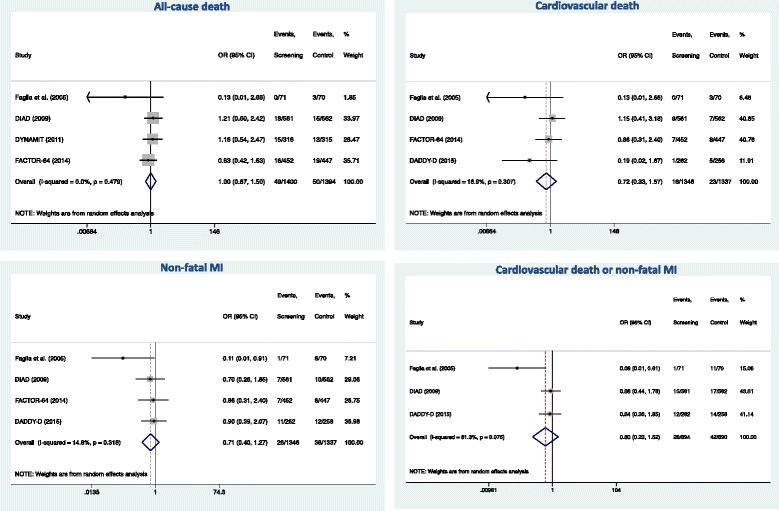
Effect of screening on all-cause death, cardiovascular death, non-fatal myocarial infarction (MI), the composite of cardiovascular death or non-fatal MI. Odds ratios (OR) with 95 % confidence intervals (CI). For each study, the area of the box represents its contribution to the meta-analysis (weight). Numbers of events are shown in the screening group and in the control group. Overall estimates of effects were calculated with a random effect model

Follow-up data were available in 4 studies for all-cause death, cardiovascular death, and non-fatal MI, and in three studies for the combined endpoint of cardiovascular death or non-fatal MI. The proportion of patients with events during follow-up were 3.5, 1.5, 2.4, and 3.9 %, for all-cause death, cardiovascular death, non-fatal MI, and the composite cardiovascular death or non-fatal MI, respectively. Figure 2 shows forest plots for the effect of screening on clinical events. In the pooled analysis, there was no impact of screening on all-cause death (OR = 1.00 [0.67–1.50], *P* = 0.996), cardiovascular death (OR = 0.72 [0.33–1.57], *P* = 0.407), non-fatal MI (OR = 0.71 [0.40–1.27], *P* = 0.246), and the composite cardiovascular death or non-fatal MI (OR = 0.60 [0.23–1.52], *P* = 0.280). The funnel plots (Additional file 2: Figure S1) suggested asymmetry along the treatment effect, indicating possible small-study effect.

### Coronary angiography and revascularization

**Fig. 3:**
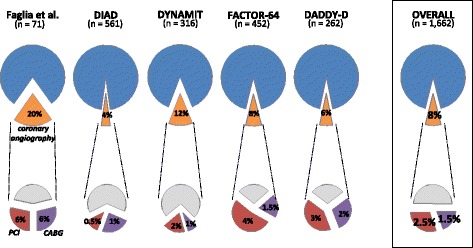
Coronary angiography and revascularization procedures in the screening arms of the 5 studies and in the pooled analysis. The proportions of coronary angiography, PCI (percutaneous coronary interventions) and CABG (coronary artery bypass surgery) are expressed relative to the number of patients undergoing screening

The results of the screening and the early protocol-related coronary procedures in the screening groups are summarized in Table 3 and Fig. 3. The number of patients with abnormal screening varied between 8 % and 21 %. The proportion of patients with abnormal screening who underwent coronary angiography varied between 22 % and 93 % (Table 3). Overall, coronary angiography related to a positive screening was performed in 130 patients out of the 1,662 patients randomized to screening (8 %). Among the 130 patients with angiography, half underwent myocardial revascularization, 40 by PCI and 25 by CABG, representing 2.5 % and 1.5 % of the total number of screened patients, respectively (Fig. 3).

**Table 3:** Results of the screening procedures for the different studies included in the meta-analysis

	Faglia et al. (2005)	DIAD (2009)	DYNAMIT (2011)	FACTOR-64 (2014)	DADDY-D (2015)
Number of patients in the screening arm	71	561	316	452	262
Patients with positive screening, n (%)	15 (21 %)	83 (15 %)	–	76 (17 %)^a^	20 (8 %)
Patients with abnormal^b^ screening, n (%)	15 (21 %)	113 (20 %)	68 (22 %)	76 (17 %)	20 (8 %)
Coronary angiography related to abnormal screening, n (%)	14 (20 %)	25 (4 %)	38 (12 %)	36 (8 %)	17 (6 %)
Proportion of patients with abnormal screening who underwent coronary angiography	14/15 = 93 %	25/113 = 22 %	38/68 = 56 %	36/76 = 47 %	17/20 = 85 %
Patients with significant CAD on coronary angiography performed subsequently to the initial screening, n (%)	9 (13 %)	9 (2 %)	–	–	12 (5 %)
Proportion of patients with coronary angiography who had significant CAD	9/14 = 64 %	9/25 = 36 %	–	–	12/17 = 71 %

^a^moderate to severe coronary stenosis by CCTA

^b^abnormal screening included patients with positive screening and patients with non-perfusion abnormality (ischemic ECG changes, transient left ventricle dilation, or baseline left ventricle dysfunction) in the DIAD study; patients with positive screening and SPECT results showing small defects (uncertain results) in the DYNAMIT study

### Impact of screening on statin, aspirin and ACE/ARB use

**Fig. 4:**
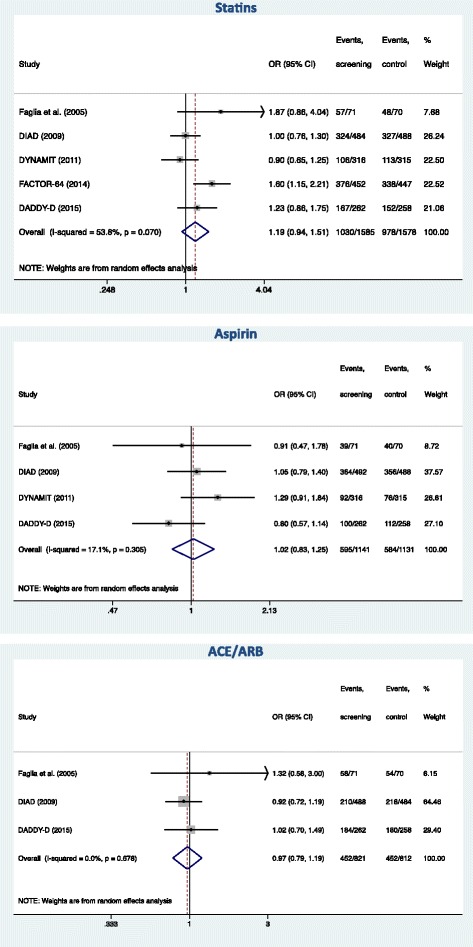
Effect of screening on statin, aspirin and angiotensin-converting enzyme inhibitor/angiotensin receptor blocker (ACE/ARB) use

Information on statins use after screening was available in all five studies. As shown in Fig. 4, there was no evidence in the pooled analysis for an increased use of statins in screened patients (OR = 1.19 [0.94–1.51] (*P* = 0.158). Information on aspirin use after screening was available in 4 studies. Likewise, as shown in Fig. 4, there was no evidence for an increased use of aspirin in screened patients (OR = 1.02 [0.83–1.25] (*P* = 0.837). Information on ACE/ARB use after screening was available in three studies. Likewise, as shown in Fig. 4, there was no evidence for an increased use of ACE/ARB in screened patients (OR = 0.97 [0.79–1.19] (*P* = 0.780).

## Discussion

A significant proportion of the diabetic patients with no clinical evidence for CAD have abnormal stress tests [8, 9, 15] and are at greater risk for cardiovascular events [16]. Screening for asymptomatic CAD in these patients is intuitively attractive as this could potentially lead to additional therapies (coronary revascularization, intensification of preventive medications) and closer follow-up. Several randomized trials have been performed to answer to this clinical question [10–14]. However, the cardiac event rates were significantly lower than originally anticipated when these studies were designed [10, 13, 14] and whether the strategy of systematic screening may improve outcome is still a matter of debate [17, 18].

The present pooled analysis shows that systematic coronary screening of asymptomatic diabetic patients has no detectable impact on the subsequent risk of all-cause death, cardiovascular death, non-fatal MI, and the composite of cardiovascular death or non-fatal MI.

In screened diabetic patients, protocol-related coronary angiographies were relatively infrequent (8 %) and myocardial revascularization was only performed in one half of this group. Although revascularization has not been shown to be superior to optimal medical therapy in the overall population of stable CAD patients with DM [19, 20], it has been suggested that the subgroup with the most severe disease may benefit from CABG [20, 21]. However, even if beneficial for some individuals, CABG in this population is very rare and its impact is unlikely to be detectable in the overall screened group. In the present analysis, CABG was performed in 1.5 % of the screened patients, a figure very similar to the 1 % rate reported in a recent prospective cohort [16].

As far as medical prevention therapy is concerned, our results show that statin, aspirin and ACE/ARB use did not differ in the screening and control groups at follow-up. This is probably related to the fact that, nowadays, diabetic patients have a good background of medical prevention irrespective of CAD diagnosis. Indeed, it is generally accepted that patients with DM have a risk approaching that of CAD patients [22] and aggressive risk factor reduction is recommended for these patients [23]. The reduced plaque burden may consequently decrease the risk of events [24].

The present study has several limitations. Firstly, even in the pooled analysis, the number of events was low and the power to exclude a difference between groups is limited. However, this demonstrates the reasonably good prognosis of asymptomatic diabetic patients candidate to screening, a useful information in clinical practice. Secondly, the study protocols differed widely. Although all included studies were designed as comparisons of screening for CAD versus no screening, the screening methods were variable (ETT, stress echocardiography, nuclear stress test, or CCTA) and have different sensitivities, specificities, and predictive values for the detection of CAD. The screening methods also provide different information for physicians (indicators of myocardial ischemia, anatomical information). It should thus be acknowledged that this is a limitation of our study, and that - if realized in the future - an adequately powered study specifically focusing on one screening method may be positive. In addition, some studies included a treatment plan in case of abnormal screening while, in other studies, the results of the screening were communicated to the personal physician or cardiologist who decided if subsequent investigations and treatments were needed. As a consequence, the rate of coronary angiography in case of positive screening varied from nearly 100 % (when indicated by the protocol) to only 20 % (when left at the physician’s decision). Regarding medical prevention, the recommendation to provide aggressive care to reduce risk factors in patients with abnormal CCTA or CAC score in FACTOR-64 is probably the explanation for the increased statin use at follow-up which was solely observed in this study (Fig. 4). Finally, the limited number of studies precluded to analyze how study-level covariates may impact on the benefit of screening.

## Conclusions

In conclusion, diabetic patients included in randomized comparisons of CAD screening versus no screening have a low risk of clinical events during follow-up. The present analysis shows no evidence for a benefit of screening in term of outcome. The proportion of patients who undergo myocardial revascularization as a consequence of screening is low; the percentage of patients undergoing CABG is in the 1 % range. Overall, screening has no detectable impact on the prescription of preventive medications including statins, aspirin and ACE/ARB.

### Ethics approval and consent to participate

Not applicable.

### Consent for publication

Not applicable.

### Availability of data and materials

The data supporting the results of this review are fully available in references [10, 14].

## Additional files

Additional file 1: Table S1. Quality assessment of the studies included in the meta-analysis. (DOCX 11 kb) Additional file 2: Figure S1. Funnel plots of studies included in the analysis on all-cause death, cardiovascular death, non-fatal myocardial infarction, the composite of cardiovascular death or non-fatal myocardial infarction. OR = Odds ratio. Se = standard error. (PDF 61 kb)

## Abbreviations

ACE: angiotensin-converting enzyme inhibitor
ARB: angiotensin receptor blocker
BMI: body-mass index
BP: blood pressure
CABG: coronary bypass surgery
CAC: coronary artery calcium
CAD: coronary artery disease
CCTA: coronary computed tomography angiography
CI: confidence interval
DM: diabetes mellitus
ECG: electrocardiogram
ETT: exercise electrocardiogram test
HbA1c: glycated hemoglobin
HDL-C: high-density lipoprotein cholesterol
LDL-C: low-density lipoprotein cholesterol
MI: myocardial infarction
OR: odds ratio
PCI: percutaneous coronary intervention
SPECT: single photon emission computed tomography
