# Supplementary material for: Screening for asymptomatic coronary artery disease in patients with diabetes mellitus: A systematic review and meta-analysis of randomized trials
Source: BMC Cardiovasc Disord. 2016 May 10;16:90. doi: 10.1186/s12872-016-0256-9 (PMC4862116; doi:10.1186/s12872-016-0256-9)
Supplement: Additional file 1: Table S1. — Quality assessment of the studies included in the meta-analysis. (DOCX 11 kb) [file 12872_2016_256_MOESM1_ESM.docx]

Supplementary Table 1. Quality assessment of the studies included in the meta-analysis.

|  | Faglia et al.  (2005) | DIAD  (2009) | DYNAMIT  (2011) | FACTOR-64 (2014) | DADDY-D (2015) |
| --- | --- | --- | --- | --- | --- |
| **Design** | Prospective  Randomized | Prospective  Randomized | Prospective  Randomized | Prospective  Randomized | Prospective  Randomized |
| **Period of inclusion specified** | Yes | Yes | Yes | Yes | Yes |
| **Control of confounding factors** | Adequate | Adequate | Adequate | Adequate | Adequate |
| **Clear description of inclusion/**  **exclusion criteria** | Yes | Yes | Yes | Yes | Yes |
| **Clear definition of endpoints** | Yes | Yes | Yes | Yes | Yes |
| **Minimization of selection bias** | Yes  Type 2 diabetic patients with at least 2 other risk factors | Yes  Type 2 diabetic patients | Yes  Type 2 diabetic patients with at least 2 other risk factors | Yes  Type 1 or type 2 diabetic patients | Yes  Type 2 diabetic patients with high cardiovascular risk score |
