# Supplementary figures and images for: Screening for asymptomatic coronary artery disease in patients with diabetes mellitus: A systematic review and meta-analysis of randomized trials
Source: BMC Cardiovasc Disord. 2016 May 10;16:90. doi: 10.1186/s12872-016-0256-9 (PMC4862116; doi:10.1186/s12872-016-0256-9)

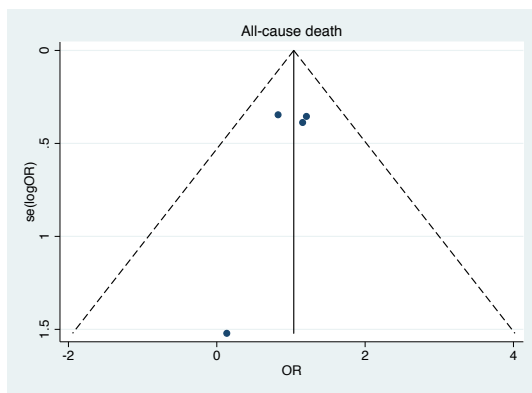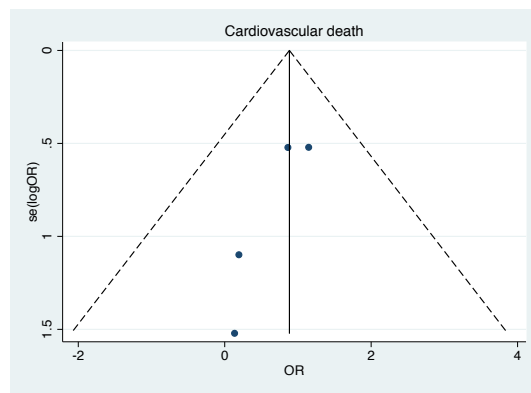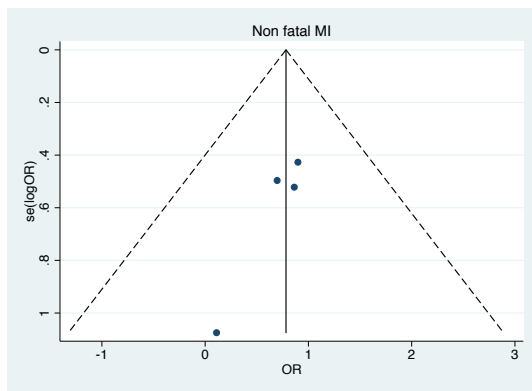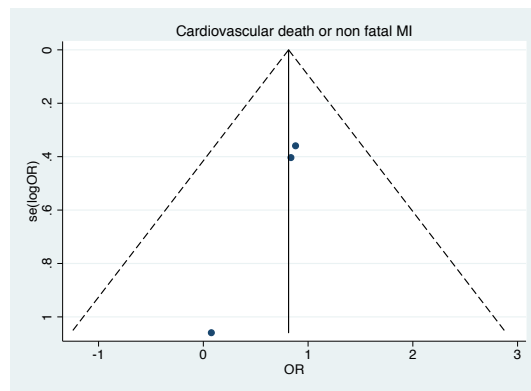

Supplement: Additional file 2: Figure S1. — Funnel plots of studies included in the analysis on all-cause death, cardiovascular death, non-fatal myocardial infarction, the composite of cardiovascular death or non-fatal myocardial infarction. OR = Odds ratio. Se = standard error. (PDF 61 kb) [file 12872_2016_256_MOESM2_ESM.pdf]
